# State and Future Science of Opioids and Potential of Biased-ligand Technology in the Management of Acute Pain After Burn Injury

**DOI:** 10.1093/jbcr/irad004

**Published:** 2023-01-13

**Authors:** David M Hill, Erik DeBoer

**Affiliations:** Clinical Pharmacist, Department of Pharmacy, Regional One Health, 877 Jefferson Avenue, Memphis, Tennessee 38103, USA; Trevena, Inc., USA

**Keywords:** Burns, Analgesics, Opioid, Pain, Adverse Drug Event

## Abstract

Pain associated with severe burn injury is one of the most intense and clinically challenging to manage, as the metabolic imbalances associated with the inflammation caused by the injury and treatment interventions (e.g., dressing changes and debridement, excision, and grafting) can further worsen the pain. In the pharmacologic management of a complex, hospitalized patient with burn injuries, opioid therapy remains an efficacious mainstay of treatment. However, the complex nature of pain, injury characteristics, and common demographics after burn injury place patients at high risk of opioid-related adverse events. Thus, guidelines recommend that decisions about choice of opioid be based on physiology, pharmacology, and physician experience, in addition to individualizing initial treatment with subsequent continual adjustments throughout care. Although substantial progress has been made in pain management strategies with utilization of nonopioid medications and nonpharmacologic adjuncts to opioid pharmacotherapy, there is still a need to evaluate new therapies, as an optimal regimen still lacks significant evidential support. Herein, we review the actions of opioids at the cellular level, contributing to both nociception and opioid-related adverse events. We also discuss the most recently approved intravenously administered opioid, oliceridine, developed utilizing biased ligand technology, including a summary of its clinical efficacy and safety in the management of severe acute pain. While oliceridine has been evaluated for the management of moderate-to-severe acute pain, the large phase 3 studies did not include patients with burn injuries. However, potential implications and future study direction for pain associated with burn injury are discussed.

## INTRODUCTION

Pain caused by acute, severe burn injury is one of the most intense and clinically challenging to manage, as the metabolic imbalances associated with the inflammation caused by the injury and treatment interventions (eg, dressing changes and debridement, excision, and grafting) can further worsen the pain. Part of the challenge is the involved complexity. After acute burn injury, both injured tissue and adjacent nonburned tissue upregulate response to painful and non-painful stimuli (hyperalgesia and allodynia, respectively).^1^ The primary mechanism is believed to be nociceptive, but is interwoven with aspects of somatogenic, neuropathic, and psychogenic pathways.^2,3^ Throughout the patient’s course of treatment, this has been described in several categories: Background pain that occurs from the burn injury itself while the patient is otherwise at rest, breakthrough pain (ie, a transient exacerbation of pain), and evoked or procedural pain (ie, associated with wound manipulation such as routine debridement and dressing changes). As such, each patient’s trajectory is unique, necessitating customized management while caring for patients with acute burn pain. In addition to the primary injury, severe burns also may require use of autografts to facilitate appropriate healing and function, complicating and intensifying the patient’s discomfort, given donor sites are often more painful than the burned tissue because underlying dermal nociceptors are often largely intact and nerves newly exposed after harvesting.^4^

Over the past several years, the burn community has employed increasingly sophisticated methods to enhance pain control through pharmacologic and nonpharmacologic means. Such methods have included distraction,^5^ music therapy,^6^ aromatherapy,^7,8^ acupuncture,^9^ oral and intravenous (IV) ketamine, and bupivacaine.^10–13^ The goal of this work has been to expand the multimodal armamentarium and to manage acute pain while minimizing the known, long-term negative consequences of poorly managed pain such as post-traumatic stress disorder and the development of chronic pain.^14,15^ The truth is that there are few options known to date to completely control all the involved aspects of acute burn pain and offer relief to the affected patients.^2^ While the field continues to make substantial progress in evolving nonopioid pain therapies, opioid therapy remains an efficacious and necessary component in the pharmacologic management of a hospitalized burn patient.^2,16–18^ Opioids carry their own risks, including dependence, tolerance, and hyperalgesia in addition to inherent side effects,^1^ and a benefit-risk assessment needs to be made for the patient while considering opioid class effects including risks of respiratory depression, addiction, abuse, and misuse. Advances in the treatment of pain with the use of opioids; however, have been limited, presenting opportunities for additional investigation.

Recently published guidelines recommend that decisions about the choice of narcotic be based on the physiology, pharmacology, and prescriber’s experience. Furthermore, the guidelines also recommend *“opioid therapy should be individualized to each patient and continuously adjusted throughout their care due to the heterogeneity of individual responses, adverse effects, and the narrow therapeutic window of opioids (Level D)*”.^2^ In practice, the profound physiological changes accompanying major burn injury, including hypovolemia, edema, and hypoalbuminemia during the first 48 hours, followed by a hyperdynamic state with high blood flow in the kidneys and liver beyond 48 hours, contribute to altered pharmacokinetic and pharmacodynamic responses to many drugs,^19^ including opioids,^16,20^ and burn patients often require higher-than-recommended doses of opioid analgesics for adequate pain relief.^18,21^ High requirements, combined with lingering active metabolites and dangerous drug–drug interactions, place patients with burn injuries at a higher risk of developing opioid-related adverse events (ORAEs) such as nausea, vomiting, respiratory depression, constipation, sedation, and pruritus. This concern is further heightened for patients at the extremes of ages or frail, have high body mass index (BMI) or are obese, or have pre-existing comorbidities.^22–25^ Post-operative patients experiencing a single ORAE require 47% higher healthcare utilization, including resources, costs, and length of stay.^26,27^

Burn patients are often treated with opioids for several days or even weeks depending on the severity and extent of their injuries which may present additional therapeutic challenges. Both injured tissue and adjacent non-burned tissue upregulate responses to painful and non-painful stimulus over time and generate hyperalgesia or allodynia.^1,28,29^ Moreover, pruritus is very common during the wound healing process after a burn, with a reported prevalence of 80%–100% that worsens healing, further complicating the recovery process and lowering the quality of life.^30^

An additional consideration for burn patients treated with opioids for extended periods is the development of tolerance, wherein the dose–response of a given opioid therapy will decrease over time, effectively elevating the dose requirements to achieve a similar analgesic benefit.^21^ Apart from the cost consequences of additional therapy, analgesia and ORAEs may not exhibit tolerance at the same rate, leaving patients proportionally more prone to adverse effects (AEs) as tolerance develops.^31^ Tolerance is often addressed clinically by using an opioid which potentiates a slightly different portion of the mu opioid receptor (mOR) pathway.^32^ Other strategies include using low doses of naloxone or buprenorphine to aid in receptor availability^33,34^; however, these options add unpredictability and further complicate pain control and ORAE management.^3,35^

**Figure 1:**
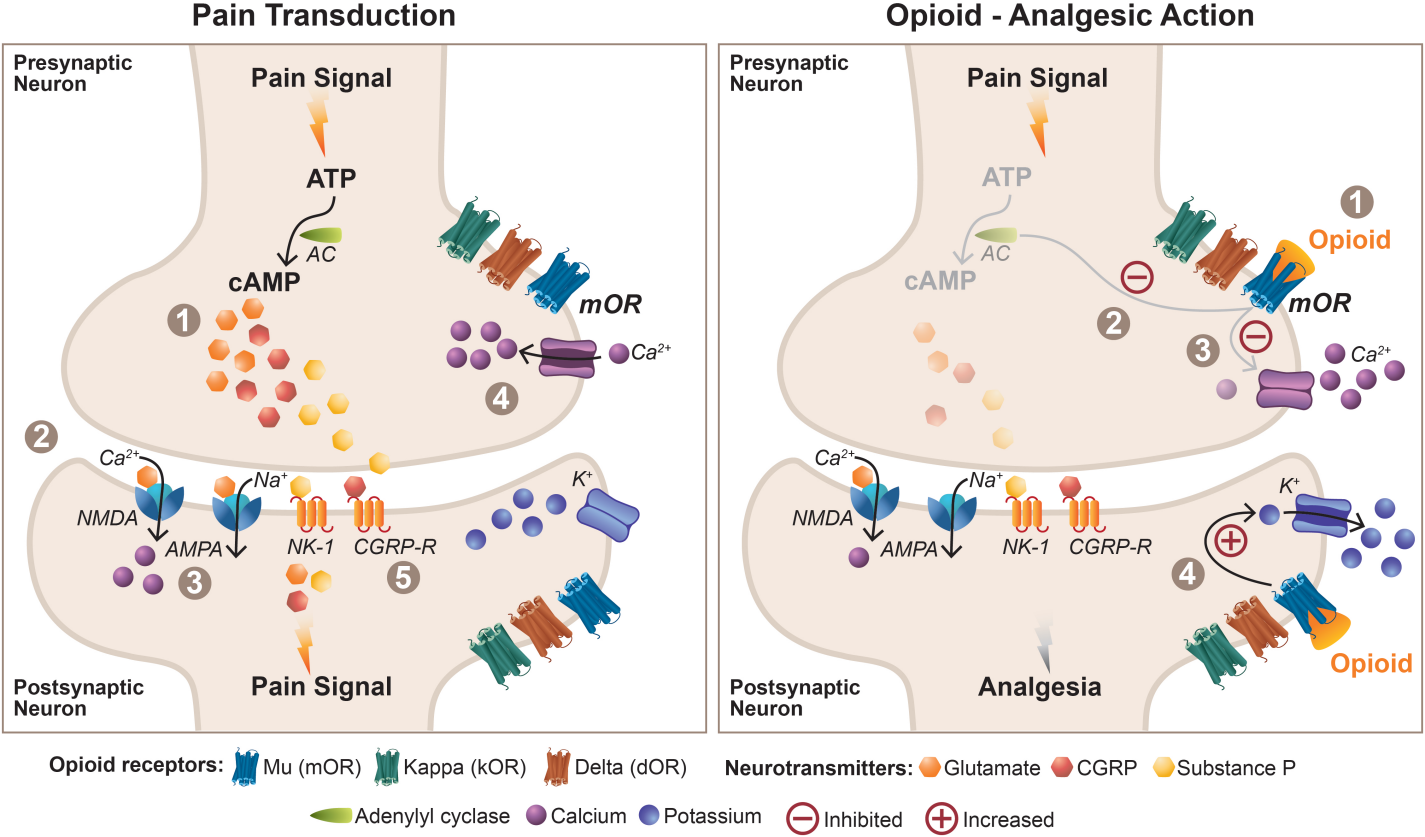
**Schematic depiction of pain transduction (*left panel*) and analgesic mechanism of action of opioids on the pain pathway (*right panel*).** (*Left panel*) Pain pathway: Following activation of nociceptors, **1)** signal transduction in pre-synaptic neurons results in activation of adenylyl cyclase and cyclic adenosine monophosphate (cAMP)-mediated release of neurotransmitters (glutamate, substance P, calcitonin gene-related protein [CGRP]) into the synaptic cleft; **2)** released neurotransmitters bind to specific post-synaptic receptors: glutamate to the α-amino-3-hydroxy-5-methyl-4-isoxazolepropionic acid (AMPA) and N-methyl-D-aspartate (NMDA) receptors, substance P to the neurokinin-1 (NK-1) receptor, and CGRPs to the CGRP receptors (CGRP-R); **3)** Activation of AMCP and NMDA receptors leads to an increase in the flow of positively-charged ions such as Ca2+ and Na2+ and increase in pain signal; another positive ion, Mg2+, is mobilized following NK-1 receptor binding-mediated protein kinase C activation and acts indirectly by unblocking NMDA receptors; **4)** Substance P also mediates an increase in the influx of Ca2+ into the presynaptic neuron, contributing to increased neurotransmitter release and signaling; **5)** Although the mechanism is not completely clear, activation of CGRP-R(s) is also believed to contribute to neuromodulation in the periphery similar to its involvement in migraine headache pathophysiology. (*Right panel*) Opioid Analgesic Action: Four subtypes of opioid receptors are present on both pre- and post-synaptic neurons: mu (µ) opioid receptor (mOR), kappa (κ) opioid receptor (kOR), delta (δ) opioid receptor (dOR), and nociception-related G protein-coupled receptor (*non-naloxone-binding; not shown in figure*). **1)** Both endogenous and exogenous opioids bind to the mOR, resulting in a series of actions that inhibit pain signaling; **2)** Activation of mOR results in an inhibition of adenylyl cyclase, leading to an inhibition of cAMP release and subsequent inhibition of neurotransmitters; **3)** mOR binding also inhibits voltage-gated Ca2* channels and influx of positive ions into the pre-synaptic neuron, contributing to an inhibition of neurotransmitter release; **4)** Finally, binding of opioids to the mOR on the post-synaptic neuron results in an opening of G protein-gated K+ channels, facilitating efflux of K+ ions and hyperpolarization leading to decreased sensitization to pain signaling.

Tolerance develops in part due to the activity of intracellular effectors downstream of the mOR. mORs are G-protein-coupled receptors (GPCRs), where G-protein activation results in inhibition of adenylyl cyclase and activation of phospholipase C. This serves to carry out the analgesic cascade of µ agonism by reduction of cyclic adenosine monophosphate and release of substance P as well as the inhibition of voltage-dependent Ca2+ channels and activation of inwardly rectifying K+ channels (Figure 1).^36^ After repeated signaling, a group of GPCR kinases known as the “arrestin” family, phosphorylate the mOR, blunting additional G-protein-coupled analgesic signaling through an internal feedback loop.^37^ Over time, receptor internalization or endocytosis occurs, reducing the presence of mOR at the post-synaptic surface through a beta arrestin-mediated mechanism.^38^ In this way, with repeated signaling of the mOR, it becomes less effective (desensitization) over time due to agonism of the endogenous arrestin pathway.^39^ When fewer mORs are present at the post-synaptic terminal the ability of a µ agonist to elicit its analgesic effects is proportionally lower, effectually desensitizing the system to µ agonism.

Few animal burn pain models have demonstrated differential opioid efficacy. Existing guidelines are supported with low quality studies and graded levels of evidence.^22^ However, evaluating drug-specific differences in a clinical setting of acute burn pain is difficult.^18^ As such, findings from other pain models are extrapolated to this setting. This review highlights an historical account of opioid use and turns attention to a next generation of µ-opioid agonist, oliceridine, and its potential to aid patients and providers caring for patients with burn injuries.

## Opioids: Historical Compounds

**Figure 2:**
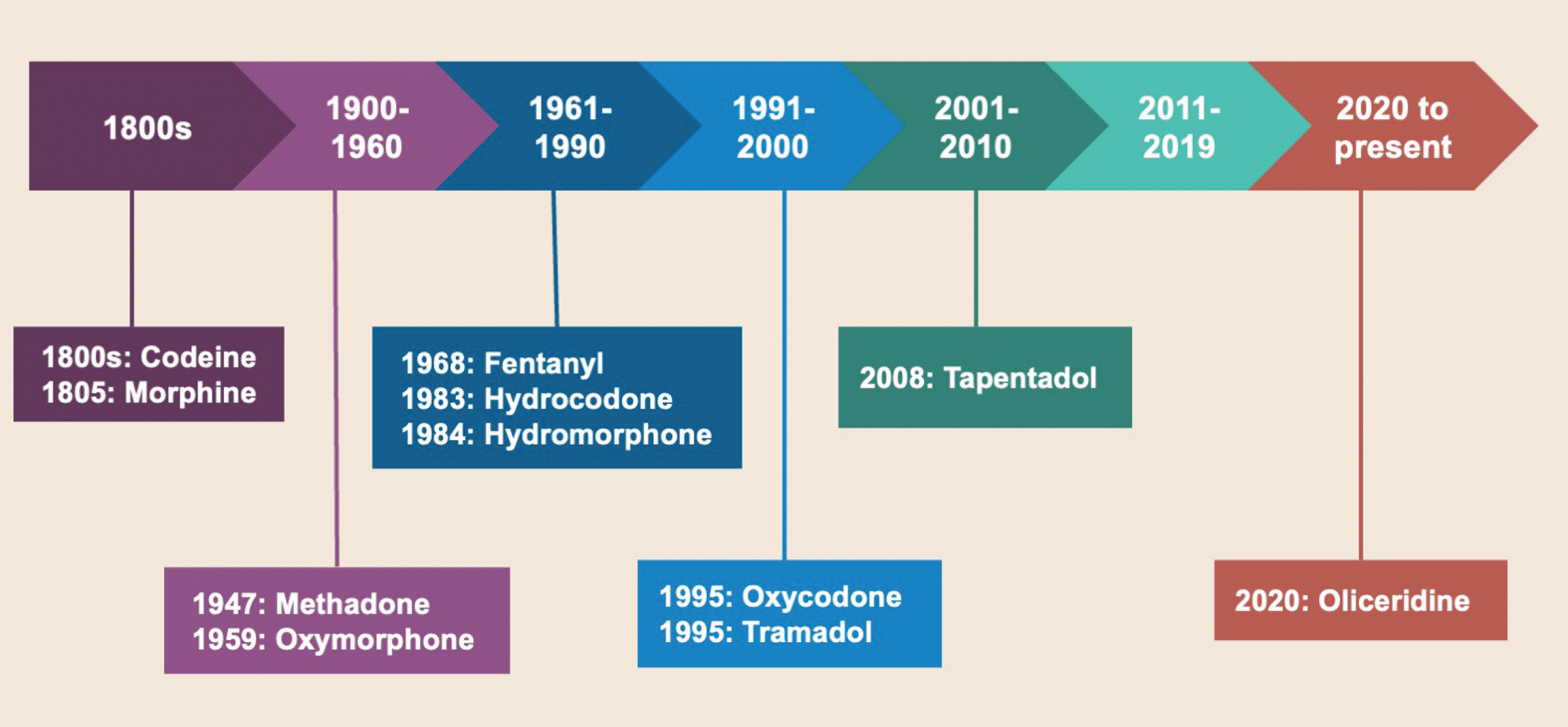
Timeline of Availability of Opioids.

Opioids (refers to opiates, the natural products obtained from the opium poppy, as well as semi-synthetic or synthetic opioids) are ubiquitous in the management of pain.^18,40^ Morphine was the first opium available in the early 1800s, followed by codeine (which although found in opium, can also be synthesized). Their widespread use led to the original Pure Food and Drug Act of 1906—the first piece of U.S. federal legislation regulating the pharmaceutical marketplace.^41^ The first synthetic opioid medications were developed in the 1910s, with the availability of methadone in 1947^42^ and oxymorphone in 1959. In the 1960s and 1970s, the US Food and Drug Administration (FDA) approved short-acting agents such as fentanyl and combination products such as oxycodone/acetaminophen (1976). In the late 1980s and early 1990s, the FDA approved additional semi-synthetic opioids (eg, hydromorphone, hydrocodone, oxycodone, tramadol), combination products, and long-acting formulations, such as morphine sulfate controlled-release (MS Contin^®^ in 1985) and oxycodone extended-release (ER) (1995). Starting around 2008-2011, the FDA has approved a cohort of opioids, including tapentadol and other ER compounds with abuse-deterrent properties (tapentadol ER, hydromorphone ER) (Figure 2).^41^ Oliceridine was approved in 2020 for the management of acute pain severe enough to require an IV opioid analgesic and for whom alternative treatments are inadequate.

### Actions of Opioids at the Cellular Level

Exogenous opioids mimic and potentiate the physiologic action of endogenous opioids, through their activity on opioid receptors.^36^ The opioid system consists of four receptor subtypes, including, mu (µ), kappa (κ), delta (δ), and nociception that are GPCRs sharing the similar seven-transmembrane topology. The nociception-opioid receptor (nOR) subtype does not bind naloxone, nor are its effects reversed by naloxone, and is considered a non-classical opioid receptor. Classical opioid receptors are expressed widely within the central nervous system and, to a lesser extent, throughout the periphery tissues (including skin, joints, viscera).^43,44^ The analgesic effects of opioids are elicited by central activation of opioid receptors; however, many of the AEs (eg, gastrointestinal (GI) motility, urinary retention, and pruritus) are regulated by activation of peripherally located opioid receptors.^45^

### Opioid Effects by Receptor Subtype

In addition to contributing predominantly to the analgesic effect, mORs are responsible for respiratory depression, euphoria, sedation, reduced GI motility, and physical dependence.^46^ Activation of the κ opioid receptors (kORs) can cause sedation, dyspnea, dependence, dysphoria, respiratory depression, and spinal analgesia,^44,46^ while δ opioid receptor (dOR) activity modulates rewarding activities of a number of drugs of abuse, and influences several aspects of addictive behaviors including drug-seeking, emotional responses, and learning processes.^43,47^ dORs co-localize with mORs in enteric neurons, and also contribute to reduced GI motility.^48^ Data from animal studies show that mOR and dOR activation causes respiratory depression as a result of decreased breathing frequency, and kOR activation causes an increase in tidal volume.^49^ The most common effects by opioid receptor subtypes are shown in Table 1. An image to be recounted later, it is important to conceptualize that opioids can be selective to a particular receptor, but still have activity at other GPCRs. For example, morphine has a higher affinity for mOR of the GPCR, but also retains differing levels of binding capacity to kOR and dOR.^50–52^ All opioids are not necessarily created equally.

**Table 1:** Most common effects of opioids based on activation of receptor subtypes

Mu (mOR)	Kappa (kOR)	Delta (dOR)
Analgesia	Analgesia	Analgesia
Respiratory depression	Respiratory depression	Respiratory depression
Sedation	Sedation	Affective behavior
Miosis	Miosis	Reinforcing actions
Euphoria	Dysphoria	Reduced GI motility
Reduced GI motility	Hallucinations	
Physical dependence	Physical dependence	

Note: *dOR*, delta opioid receptor; *GI*, gastrointestinal; *kOR*, kappa opioid receptor; *mOR*, mu opioid receptor.

Although, one or more receptor subtypes can be involved with analgesic effects and AEs, multiple mechanisms could mediate differential *in vivo* activities of drugs targeting opioid receptors.^53^ Indeed, factors such as intrinsic drug efficacy, pharmacogenetics,^54–56^ pharmacodynamics, drug selectivity, and accessibility to selective receptor populations may contribute to differential effects of many opioid agonists.^53^ For example, fentanyl is highly lipophilic, facilitating rapid diffusion through membranes, including the blood brain barrier, resulting in a rapid onset of action,^43^ whereas morphine has a comparatively low lipid solubility resulting in a relatively slower penetration of the blood brain barrier and onset of action.^43^ The analgesic effect of morphine, the archetypal opioid analgesic, is mainly caused by its metabolite, morphine-6-glucuronide (M6G) instead of the parent compound.^57^ In preparation for subsequent renal elimination, morphine undergoes metabolism in the liver to morphine-3-glucuronide (M3G) (90%) and M6G (10%) by UDP-glucuronosyltransferases (specifically UGT2B7).^58^ Although lacking analgesic efficacy, the major metabolite (M3G) should not go unconsidered. M3G is centrally active and attributed to many unfavorable effects related to behavior, dependency, hyperalgesia, and potentially even opposing analgesia.^59^ Both M6G and M3G accumulate in renal insufficiency. The accumulation of M6G in patients with renal insufficiency has been implicated in morphine toxicity, and the dose of morphine should be adjusted according to the patient’s current kidney function.^57,60^ Structural differences between opioids also contribute to differences in metabolism.^61^ As previously mentioned, patients’ genetic polymorphisms can vary their ability to metabolize different opioids, accordingly. Altered metabolism can either lead to opioid or metabolite being excreted too rapidly, not reaching its therapeutic effect, or prolonged elimination, producing toxic effects.^61^ Increased potency and selectivity at the mOR can also contribute to the likelihood of overdose and abuse potential.^62^

### Multi-receptor Ligands

Focusing on the search for opioids with improved side effect profile and low abuse potential, several novel approaches have emerged. One such approach has led to the development of multi-opioid receptor ligands, simultaneously targeting mOR/kOR, mOR/NOR or mOR/DOR.^63^ Although compounds simultaneously targeting mOR and kOR, either as agonist/agonist or agonist/antagonist, have shown some clinical efficacy, the limitations associated with the mOR activation, such as potential abuse liability, constipation, and respiratory depression, still remain.^63^ Most progress has been made in the development of mOR/NOR bifunctional agonists. Compounds with affinity for both NOR and mOR are under development in the “proof of concept” stage. These compounds may have a useful profile as analgesics with a reduced addiction liability and a slower development to tolerance.^63^

### Biased Agonism

Much of the knowledge of GPCR inner workings comes from decades of groundbreaking work from groups like Drs. Lefkowitz and Kobilka.^64–67^ Work so profound, their group was awarded the Nobel Prize in Chemistry in 2012.^68,69^ As described earlier, in addition to signaling through G proteins, GPCRs can also activate G-protein-independent signaling pathways through multi-functional adaptor proteins called arrestins. GPCRs exist in multiple conformational states, where each conformation confers different downstream effects.^37^ While the downstream signaling cascades of G-protein contribute to antinociception, ubiquitously expressed arrestins, particularly arrestin-3 (β-arrestin2), play a role in GPCR internalization and desensitization, diminishing G-protein-mediated signaling.^37,70^ Following activation, GPCRs are phosphorylated by members of the family of G-protein–coupled receptor kinases (GRKs). Phosphorylated receptors are then bound by arrestins, which prevent further stimulation of G proteins and downstream signaling pathways.^71^ In addition, the GRK-arrestin system facilitates GPCR internalization of inactivated receptors, reducing the presence of mOR at the post-synaptic surface through a beta arrestin-mediated mechanism.^71^ Through this mechanism, repeated signaling of the mOR becomes less effective over time, due to agonism of the endogenous arrestin pathway.^39^ β-arrestin-2 is also known to regulate GI, respiratory function, and subjective effects (ie, liking, dependence, and withdrawal); however, the mechanisms are unknown.^70^

**Figure 3:**
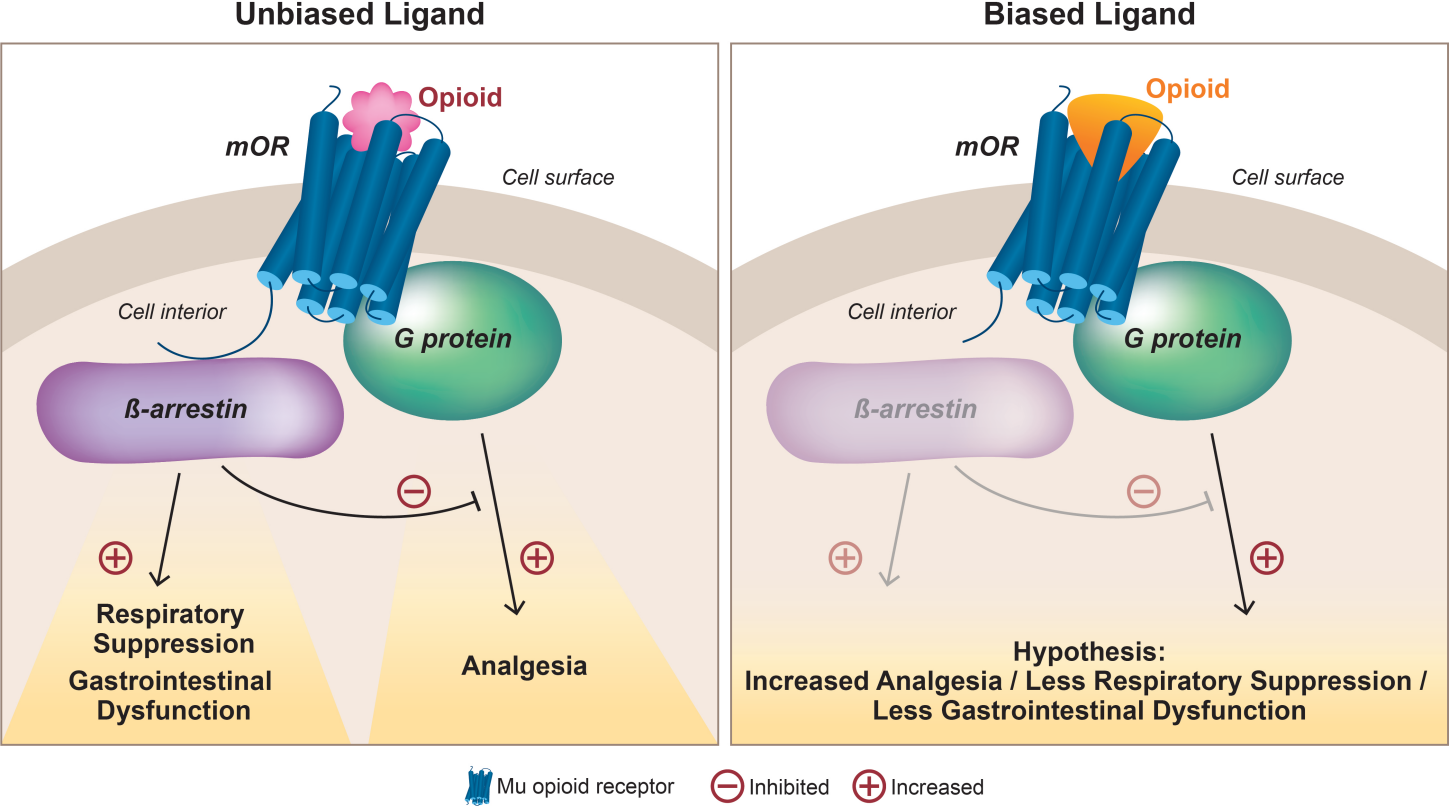
**Hypothesis for the G Protein-Biased Ligands at the Mu-opioid Receptor.** Unbiased or non-biased agonists (ligands) can efficiently activate both G protein- and β-arrestin-dependent signaling. In contrast, biased agonists at the mu opioid receptor (mOR) preferentially activate G-protein signaling and may offer increased analgesia with improved safety and tolerability.

The concept that biased agonists at the mOR might provide a therapeutic advantage favoring effective analgesia stems from observations in β-arrestin-2 knockout mice.^72,73^ In β-arrestin-2 knockout mice, morphine was less prone to receptor desensitization and tolerance, providing increased analgesia and causing less respiratory depression and constipation.^72,73^ Such findings supported efforts to design agonists with bias toward G-protein-mediated signaling which may result in improved therapeutic index (Figure 3).

Biased agonism can also occur at other GPCRs, such as DOR and kOR. It has been postulated that G-protein–biased agonists of the DOR may offer an approach to develop analgesics for chronic pain states and therapeutic agents to treat emotional disorders such as depression with a reduced AE profile.^74^ Likewise, G-protein-biased kappa agonists are hypothesized to reduce pain and itch, with fewer side effects such as anhedonia and psychosis; and research is underway exploring the potential development of G-protein-biased kappa agonists as adjuvant analgesics with reduced addictive potential.^75^ The research in the biased-ligand technology focusing on the mOR is much more advanced than the DOR and kOR. Oliceridine is the first G-protein agonist at the mOR, developed utilizing biased-ligand technology,^70^ and is currently FDA approved for the management of acute pain severe enough to require IV opioid analgesia.

## Oliceridine

### Nonclinical Data

TRV130, or oliceridine, was discovered as a result of high-throughput screening from a chemical library.^70^ In human embryonic kidney cells, oliceridine was found to be more potent for G-protein stimulation (EC_50_ = 8 nM vs. 50 nM for morphine) but with only 14% of the efficacy of morphine for β-arrestin-2 recruitment, correlating with minimal mOR internalization.^50^ Additionally, cell-based assays revealed oliceridine to be approximately 400-fold selective for the mOR than the kOR, DOR, and NOR, while morphine was only 10-fold selective for the mOR over the kOR and DOR subtypes.^50^ Oliceridine demonstrated approximately 3-fold preference for the G-protein pathway over β-arrestin-2 relative to fentanyl.^50^*In vivo* studies in mice models showed oliceridine is a potent analgesic and causes less GI dysfunction and respiratory suppression than morphine at equianalgesic doses.^50^ In the animal models, the antinociceptive effect of oliceridine can be antagonized by the opioid antagonist naloxone. Another study, using a murine model, reported less tolerance and opioid-induced hyperalgesia with oliceridine as compared to morphine.^76^ However, a study utilizing repeated administration of oliceridine showed constipating effects in mice and abuse potential similar to that of morphine and other mu-opioid agonists.^77^

Recent research utilizing cell-based assays or mutant strains, different than those used by DeWire et al.^50^ found oliceridine to have a low intrinsic efficacy and attributed the favorable therapeutic window and reduced AEs (eg, respiratory depression) to partial agonism, similar to that observed with buprenorphine.^78^ In a re-analysis of these findings, Stahl and Bohn clarified that the determination of “intrinsic efficacy” or “biased agonism” is dependent on the cellular signaling systems (ie, receptor density, effector expression, and amplification of signal), as well as reference agonist used; and the differing cellular systems can contribute to the diverse signaling profiles.^79^ These authors concluded that oliceridine is a biased agonist for G protein signaling through partial agonism in both G-protein signaling and β-arrestin-2 recruitment with the intrinsic efficacy uncoupled from biased agonism, contributing to the improved therapeutic window.^79^

### Clinical Pharmacokinetics

When administered IV over 1 minute to 1 hour, oliceridine exhibited a half-life (t½) of approximately 1.5 to 3 hours.^80^ The mean steady-state volume of distribution of oliceridine ranges between 90-120 L, indicating extensive tissue distribution, and the plasma protein binding of oliceridine is 77%. Oliceridine undergoes extensive hepatic metabolism by both cytochrome P450 (CYP) enzymes CYP2D6 and CYP3A4 with each enzyme contributing equally.^80^ Importantly, none of the metabolites are pharmacologically active.^80^ Mean oliceridine clearance in subjects with end stage renal failure (49.2 L/h) was 81.2% of that observed in healthy subjects (55.3 L/h), demonstrating doses do not need to be adjusted in patients with renal impairment.^81^ The volume of distribution increases with liver dysfunction. Although no dose adjustment is necessary for patients with mild or moderate hepatic impairment, patients with severe impairment may require an initial dose reduction followed by fewer subsequent doses of oliceridine, as a longer half-life is observed.^81^

### Early Clinical Studies

An early phase 1 study of oliceridine in healthy human volunteers, compared to morphine, showed superior analgesia in experimental cold pain test (CPT).^82^ Oliceridine, administered IV at bolus doses of 1.5 mg, 3 mg, and 4.5 mg elicited rapid and significant increases in CPT hand removal latency from baseline with peak efficacy 10 minutes post-dose. The geometric means were 81, 106, and 116 seconds for oliceridine (1.5 mg, 3 mg, and 4.5 mg, respectively), vs 41 seconds for placebo (*p* < .0001), and 75 seconds for morphine 10 mg (*p* < .02 vs oliceridine 3 mg and 4.5 mg). Oliceridine 1.5 mg IV produced similar analgesia as morphine 10 mg IV.^82^ All doses of oliceridine had a significantly lower effect on respiratory drive (as measured by ventilatory response to hypercapnia) than morphine (−7.3, −7.6, and −9.4 h·min/L for oliceridine 1.5, 3, and 4.5 mg, respectively, vs −15.9 h·min/L for morphine 10 mg; each *p* < .05 vs morphine). Incidence of nausea was also less with oliceridine than morphine.^82,83^

### Phase 2 Studies

In a phase 2 randomized, double-blind, adaptive-design, fixed-dose study in patients experiencing post-operative pain after bunionectomy, median onset of analgesia was 1-2 minutes with oliceridine 2-3 mg IV after the first dose (6 minutes with morphine 4 mg IV), as measured by two-stopwatch method.^84^ Meaningful pain relief with oliceridine occurred in under 5 minutes. No serious adverse events (SAEs) were reported. The most frequent adverse reactions reported in all active treatment groups were dose-dependent nausea, dizziness, headache, and vomiting.^84^

A phase 2b study conducted in patients undergoing abdominoplasty utilized a patient-controlled analgesia (PCA) regimen: two 0.75 mg loading doses of oliceridine separated by 10 minutes, followed by 0.1 mg demand doses, increased to 0.35 mg, compared to morphine PCA treatment regimen consisting of two 2 mg loading doses separated by 10 minutes followed by 1 mg demand doses.^85^ Similar analgesia was observed with oliceridine and morphine (vs placebo), with lower incidence of nausea (41% and 46% with oliceridine 0.1 mg and 0.35 mg demand dose, respectively, vs 72% with morphine; *p* < .01) and vomiting (15% with both oliceridine demand dose regimens vs 42% with morphine; *p* < .01).^85^ Additionally, respiratory events (hypoventilation and respiratory depression) were also significantly lower with both oliceridine regimens (15% with 0.1 mg demand dose, and 31% with 0.35 mg demand dose) vs morphine regimen (53%, *p* < .05 for both oliceridine regimens vs morphine).^85^

### Phase 3 Studies

The three pivotal phase 3 studies, including two randomized placebo- and morphine-controlled trials (APOLLO studies)^86,87^ and an open-label, safety study (ATHENA)^88^ were key to the FDA approval of oliceridine for use in severe acute pain. These studies evaluated oliceridine in over 1,500 patients with moderate-to-severe acute pain; however, no patients with burn injury were enrolled.

#### Randomized controlled clinical studies

In the two randomized, double-blind, placebo- and morphine- controlled studies in patients with moderate-to-severe acute pain following either orthopedic surgery-bunionectomy (APOLLO-1; *N* = 389) or plastic surgery-abdominoplasty (APOLLO-2; *N* = 401),^86,87^ patients with a numeric (pain) rating scale (NRS 0 to 10; 10 being the worst pain imaginable) score ≥4 (bunionectomy) or ≥5 (abdominoplasty) were randomized to receive one of five demand-dose (PCA) regimens: oliceridine 0.1 mg, 0.35 mg, or 0.5 mg; a morphine-control of 1 mg; or a volume-matched placebo dose. Patients were treated for up to 48 hours in APOLLO-1 and for up to 24 hours in APOLLO-2. The loading dose for all oliceridine treatment regimens was 1.5 mg; demand doses were 0.1, 0.35, or 0.5 mg, according to assigned treatment group. Supplemental doses of 0.75 mg were permitted, beginning 1 hour after the loading dose, and hourly thereafter, as needed. The loading dose for the morphine treatment regimen was 4 mg; the demand dose was 1 mg; and supplemental doses of 2 mg were permitted, beginning 1 hour after the loading dose, and hourly after, as needed. A lockout interval of 6 minutes was used for all PCA regimens. Etodolac 200 mg every 6 hours as needed was allowed, as the rescue medication. No other multimodal analgesia was included in these studies.^86,87^ The primary efficacy end point was treatment responders (vs placebo); defined as: meeting ≥30% improvement in time-weighted sum of pain intensity difference from baseline at 48 h (APOLLO-1) or 24 h (APOLLO-2) with no use of rescue pain medications, no early discontinuation of study medication, and not reaching protocol-specified dosing limit.^86,87^ The maximum allowable dosing limit for both studies was to not exceed 60 mg in the first 12 hours.

Responder rates were 50, 62, and 65.8% in the 0.1, 0.35, and 0.5 mg regimens, respectively (all *p* < .0001 vs placebo, 15.2%) in the bunionectomy study (APOLLO-1) and 61, 76.3, and 70.0% for the three demand dose regimens, respectively, (*p* < .05 vs placebo, 45.7%) in the abdominoplasty study (APOLLO-2). The responder rate for morphine was 71.1% (*p* < .001 vs placebo) in the bunionectomy study and 78.3% (*p* < .001 vs placebo) in the abdominoplasty study. In both studies, exploratory analyses indicated that the oliceridine 0.35 mg and 0.5 mg demand dose regimens were noninferior to morphine 1 mg demand dose.^86,87^

**Figure 4:**
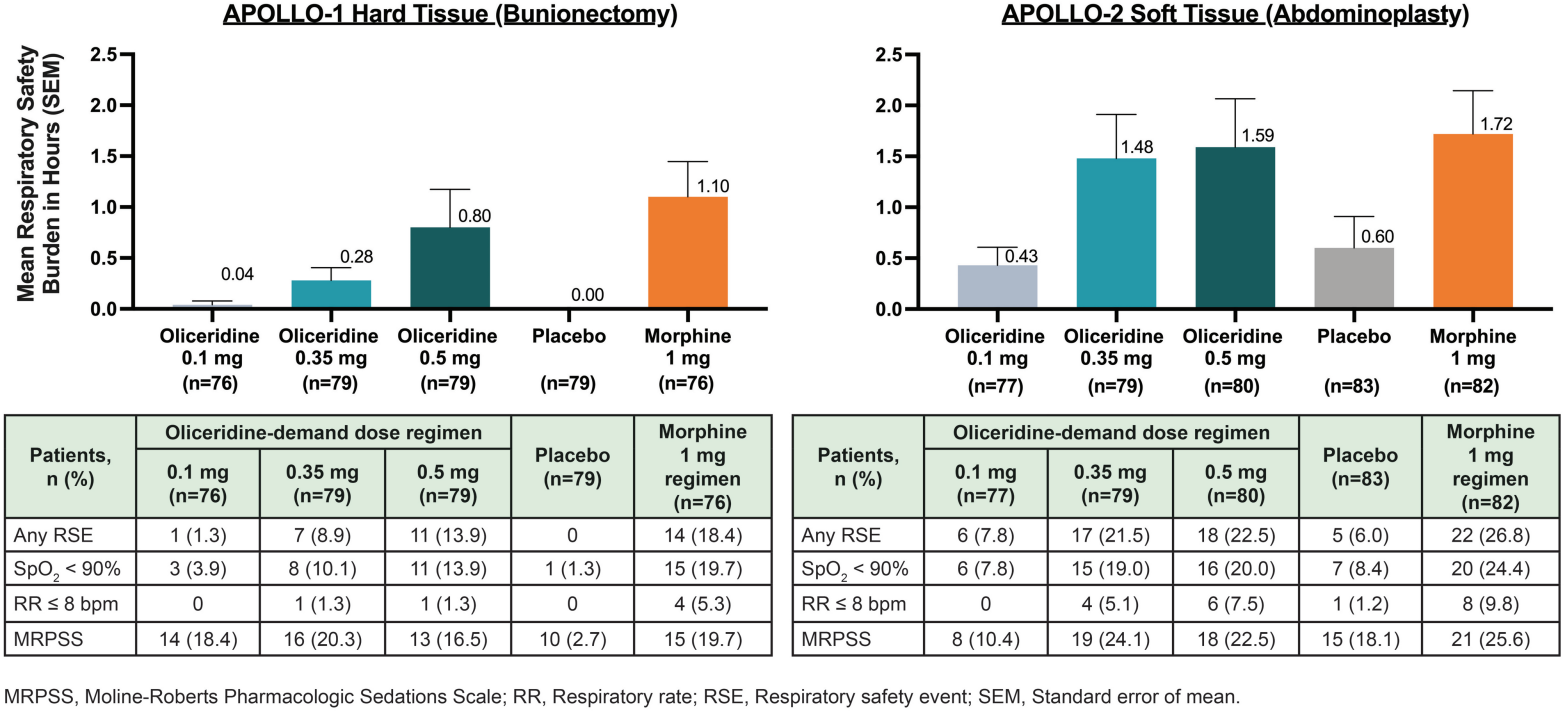
**Respiratory Safety of Oliceridine in Phase 3 Randomized Clinical Trials (Adapted from Gan and Wase 2020).** Respiratory safety burden (RSB) and respiratory safety events (RSE) in pivotal Phase 3 studies of oliceridine.^86,87^ RSB was calculated as the mathematical product of the incidence of respiratory safety events and the mean duration of such events in affected patients. No statistically significant differences for any of the oliceridine treatment groups vs. morphine. RSEs were changes in respiratory rate, oxygen saturation (SpO_2_ < 90%), and sedation measured using the Moline-Roberts Pharmacologic Sedation Scale.

A key secondary end point in both studies was the respiratory safety burden (RSB), defined as the mathematical product of the incidence of a defined set of observed respiratory safety events (RSE) multiplied by the mean expected cumulative duration of these events (in hours).^86,87^ The RSEs measured were clinically relevant changes in respiratory rate, oxygen saturation, and sedation (measured using the Moline-Roberts Pharmacologic Sedation Scale). The RSB and RSE measures were numerically lower for each oliceridine demand dose regimen compared to morphine although statistical significance was not reached (Figure 4**).** Of note, event rates were lower than *a priori* anticipation utilized to guide sample size determination, and thus enrollment was not sufficient to exclude a difference that may exist.

In both studies, where prophylactic antiemetics were not permitted, GI adverse events (nausea, vomiting) appeared to be numerically lower with oliceridine than morphine. Analyzing the pooled data (ie, improving power for statistical comparison) from both phase 3 randomized controlled studies, the incidence of nausea was lower with oliceridine 0.1 mg (40%) and 0.35 mg (59%) demand doses than with morphine 1 mg (70%) demand doses with a significant risk reduction of 43% (0.1 mg demand dose vs morphine, *p* < .001). Likewise, the incidence of vomiting was also lower with all demand doses of oliceridine: 20% with 0.1 mg, 30% with 0.35 mg and 42% with 0.5 mg, than with morphine 1 mg (52%); with a 41-61% significantly lower relative risk observed with the 0.1 mg and 0.35 mg demand dose (*p* < .001 vs. morphine).^80,89^

To further characterize the GI tolerability, an exploratory analysis evaluated the composite endpoint of complete GI response (defined as no vomiting and no use of rescue antiemetics), using a logistic regression model. In this analysis, a higher proportion of oliceridine-treated patients achieved a complete GI response than morphine-treated patients. Furthermore, after adjustment for equianalgesic conditions, the odds ratio to experience complete GI response with oliceridine (combined demand doses) vs morphine was 3.14 (95% CI: 1.78, 5.56; *p* < .0001) and 1.92 (95% CI: 1.09, 3.36; *p* = .024) in the bunionectomy and abdominoplasty studies, respectively.^89^

#### Open-label safety study

The phase 3 multicenter, open-label study, ATHENA, evaluated the safety and effectiveness of oliceridine in moderate-to-severe acute pain in post-surgical and non-surgical medical settings.^88^ A total of 768 patients aged 18 years and older (mean age 54.1 years) with surgical or non-surgical pain rated ≥4 on an 11-point NRS received at least one dose of IV oliceridine either by clinician administered bolus or PCA. The study population was mainly in a surgical setting (94%), with orthopedic (30%), colorectal (15%) or gynecologic (15%) procedures being the most common surgical types. This study also enrolled 60 patients (8%) undergoing plastic surgery, including 3 patients with wound closure unrelated to burn (i.e., 1 patient with a skin cosmetic procedure and 2 patients with skin grafting). Thirty-two percent of the patients were aged 65 years or older, 46% had a BMI ≥ 30 kg/m^2^, and each patient had at least one comorbidity. Bolus dosing was initiated at 1-2 mg with an optional additional 1 mg dose after 15 min, followed by 1–3 mg doses every 1-3 h as needed. In the emergency department and post-anesthesia care units, loading doses of 1–3 mg were followed by supplemental 1–3 mg doses every 5 min as needed. For PCA, the loading dose was 1.5 mg followed by demand doses of 0.5 mg with a lockout interval of 6 min. Supplemental doses of 1 mg were allowed. Treatment duration for each patient was based on the individual clinical need, and the protocol limited the maximal duration of oliceridine treatment to 14 days.^88^ Unlike the randomized controlled studies, prophylactic antiemetics and multimodal non-opioid analgesics were allowed and overall, 84% of patients received oliceridine as a part of multimodal analgesic regimen. The median cumulative dose was 19.3 mg, ranging from 0.9 to 223.5 mg. The median cumulative duration of exposure was 20.3 hours, ranging from 0 hours (for patients who received a single dose) to 142.7 hours (~6 days).^88^ No deaths or significant cardiorespiratory events were reported. Serious adverse events were reported in 26 patients (3%). Most SAEs were due to surgical complications, secondary to underlying medical condition. All SAEs resolved or were resolving at the time of study completion. Only 3 patients experienced SAEs determined by the investigator to be “possibly related” to oliceridine (ie, post-operative ileus, respiratory depression with respiratory rate < 8 breaths per minute, hepatic/renal failure which was confounded by surgical complications). It is noteworthy that no patients received naloxone for reversal of any respiratory event while receiving oliceridine. The most common AEs were nausea (31%), constipation (11%) and vomiting (10%) (Table 2). Pruritus incidence (an important consideration for treating patients with burn injury) was less than 5%. A good indicator of respiratory event risk, somnolence/sedation was reported in less than 2% of patients; and at the end of the study, 93% of patients self-reported “none to minimal” sedation. Opioid induced respiratory depression (OIRD), defined as oxygen saturation < 90%, occurred in 6% of patients. In an exploratory analysis of just the 724 surgical patients in the ATHENA study, the incidence of OIRD was 13.7% using a broader definition (SpO_2_ < 90% in 5.2% and respiratory rate < 10 bpm in 9.3%), and advanced age (ie, ≥ 65 years of age) and/or increased BMI (≥ 30 kg/m^2^) was not associated with increased risk of OIRD.^90^

**Table 2:** Summary of adverse events reported in the Phase 3 open-label safety trial, ATHENA (*Bergese et al. 2019*)

Parameter	All Patients *N* = 768 n (%)
Patients with at least one AE	490 (63.8)
Patients with at least one SAE	26 (3.4)
Patients with at least one AE leading to early study medication discontinuation	17 (2.2)
Possibly or probably related to study drug	256 (33.3)
Most Common AEs (≥5%)	
Nausea	239 (31.1)
Constipation	84 (10.9)
Vomiting	80 (10.4)
Pruritus	38 (4.9)
Hypokalemia	36 (4.7)
Dizziness	34 (4.4)
Headache	34 (4.4)
Hypotension	28 (3.6)
Insomnia	28 (3.6)
Pyrexia	25 (3.3)
Hypocalcemia	24 (3.1)
Hypophosphatemia	23 (3.0)
Procedural Nausea	21 (2.7)
Flatulence	20 (2.6)
Somnolence	6 (0.8)
Sedation	8 (1.0)

Note: *AE*, adverse event; *SAE*, serious adverse event.

## Implications and Future Direction

As discussed, both anecdotes and evidence-supported guidelines for managing pain in patients with burn injuries is fraught with failure. Till date, there has been no overwhelmingly positive finding to offer total relief to such a patient. There is no good answer. But there is hope. Preclinical work and the science supporting biased-ligands and oliceridine’s development bring new optimism to patients with burn injuries and providers charged with their care. Although no patient with burn injury was enrolled in the oliceridine clinical studies, findings from the phase 2 and randomized, controlled phase 3 studies have established that oliceridine is a potent analgesic. Additionally, findings from the phase 3 open-label safety study and pooled and exploratory analyses, suggest a potentially improved respiratory and GI safety profile associated with oliceridine. In addition, the low incidence rate of pruritus and somnolence/sedation observed in the phase 3 open-label safety study makes oliceridine a potentially attractive option for use in the management of pain after burn injury. With these considerations, the RELIEVE phase 4 case-controlled study (NCT05465226) is currently exploring efficacy, safety, and the pharmacokinetics of oliceridine specifically for patients with burn injuries who require opioid treatment for their acute severe pain. With continued investment in research (i.e., the RELIEVE trial) and efforts, an answer may be found to offer future patients a better solution.

## Notes

**Conflicts of Interest and Source of Funding.** There was no funding for this review. Trevena, Inc paid open access fees. DH is a consultant for Trevena, Inc. and has participated in the development of investigator-initiated trials supported by Trevena, Inc. DH is a consultant for Medline Industries, LP. EDB is an employee at Trevena, Inc.
